# In Vivo Reflectance Confocal Microscopy in General Dermatology: How to Choose the Right Indication

**DOI:** 10.5826/dpc.1002a32

**Published:** 2020-04-03

**Authors:** Chiara Franceschini, Flavia Persechino, Marco Ardigò

**Affiliations:** 1 Clinical Dermatology Department, San Gallicano Dermatological Institute (IRCCS), Rome, Italy; 2 Department of Clinical and Molecular Medicine, Sapienza University of Rome, Italy

**Keywords:** reflectance confocal microscopy, static and handled probe, melanoma, nonmelanoma skin cancer, inflammatory skin diseases, limits

## Abstract

Reflectance confocal microscopy (RCM) is a high-resolution, noninvasive imaging technique being increasingly used as an aid to diagnosis in the dermatology setting. RCM is applied in the diagnosis of both melanoma and nonmelanoma skin tumors, but also in the interpretation and management of inflammatory skin diseases. Two different devices with different designs for specific indications are available in the market: a static and a handheld probe. Several clinical presentations of the lesion could affect the examination, such as the presence of ulceration or hyperkeratosis; moreover, the anatomical site can drive the probe selection as well as the effective indication to RCM examination. In this review article, indications for the use of RCM are described in detail with a schematic approach for practical purposes.

## Introduction

Reflectance confocal microscopy (RCM) is a high-resolution, noninvasive imaging technique being increasingly used as an aid to diagnosis in the dermatology setting. RCM is applied in the diagnosis of both melanoma and nonmelanoma skin tumors, but also in the interpretation and management of inflammatory and infectious skin diseases [1–7].

### When can RCM be used in the clinical setting?

- To increase the specificity and sensitivity of diagnosis in most of the skin cancers.
- To increase diagnostic accuracy in other diagnoses.
- To guide biopsy in a suspicious lesion.
- To map the limits of some large tumors, defining margins for a correct surgical excision.
- To follow clinical and therapeutic evolution of a skin disease.

### Which is the correct RCM instrument version to be applied according to the clinical presentation and anatomical site?

The basic principle of RCM is a point source of light, which is tightly focused on a specific point in the tissue. The light is backscattered by certain tissue structures because of variations of refractive indices within the skin; specifically, melanin, hydrated collagen, and keratin are highly reflective skin components, which appear brighter on RCM images than the surrounding structures [8–10]. Only light reflected back from the tissue focus point is allowed to enter the RCM detector through a pinhole-sized spatial filter and to be processed by the dedicated software. Currently, 2 different RCM probes are available in the market, showing different designs for different indications: a static RCM (Vivascope 1500, Caliber I.D. Inc., Rochester, NY, USA) and a handheld RCM device (Vivascope 3000, Caliber I.D. Inc.).

- The traditional wide probe RCM uses a near-infrared, low-power laser beam (830-nm diode laser, power up to 35 mW) for imaging. The laser beam is scanned in a 2-dimensional grid over the skin to obtain a thin horizontal optical section, which is displayed as a grayscale image (“single RCM image”: 500 × 500 μm field-of-view). An automated stepper can be used to obtain up to 64 sequential images, making a mosaic grid of 16 × 16 contiguous images in the horizontal plane (8 × 8 mm^2^ field-of-view; named “Vivablock”). By adjusting the focal length of the laser beam, a series of consecutive single RCM images can be stacked vertically (named “Vivastack”), from the skin surface to superficial dermis, at the same point in the tissue. RCM allows dermoscopy–RCM direct correlation by clicking on a specific area of the dermoscopy image and consequently the RCM optic moves automatically to the selected site of the lesion.
- The handheld RCM has the same technical characteristics as the traditional RCM. It allows one to investigate curved anatomical sites, such as the nose or eyelids and mucous membranes, and it does not require fixation of the probe to the skin; the field-of-view is, however, limited to 1 × 1 mm^2^ and the handheld device does not use a dermoscopic picture to guide RCM imaging [11,12].

### Which are the clinical characterizations of the lesions that affect the RCM examination?

Even if the existing probes allow the examination of the majority of lesions in the majority of anatomical sites, there are limits of RCM application described in the literature [13,14]. If several limits are effectively unsolvable, in clinical practice specific solutions can be applied in order to permit an acceptable examination of the target lesion.

The typical limits are represented by hyperkeratotic and ulcerated lesions. In acral sites, RCM can be used only in a limited number of cases (Figure 4) [15]. In Table 1, possible solutions to overcome some of the limitations of RCM are explained.

## Discussion

### Melanoma and Nevi

RCM criteria for melanoma have been extensively described in the literature and are routinely used for the management of suspicious melanocytic lesions. RCM has been demonstrated to be particularly efficient in the study of flat pigmented lesions, with good performance in the differential diagnosis of atypical nevi and with significant reduction of unnecessary excision [2,4,16]. Stanganelli et al proposed RCM after 3 months of digital dermoscopy monitoring. The excision of those lesions with RCM-positive features and/or presenting major changes on digital dermoscopic follow-up could reduce the number of biopsied nevi by about 47% [17]. In the case of suspicious melanocytic lesions, to avoid sample errors, the use of static RCM for a total lesion mapping has to be preferred, when possible, to the handheld version. In the case of nodular lesions, even if RCM does not allow the evaluation of the deep cellular component, the detection of superficial RCM features characterizing melanocytic proliferations could support lesion management, assisting in the presurgical definition of the nature of the lesion.

### Nonmelanoma Skin Cancer

Also in the case of nonmelanoma skin cancer, RCM devices have to be selected according to the anatomical sites. RCM findings of basal cell carcinoma (BCC) have been widely described in the literature: dark silhouettes, bright tumor islands with peripheral palisading, cleft-like dark spaces, dendritic cells, plump-bright cells, and dilated canalicular vessels [18–22]. BCC is classified in different histological subtypes: nodular, superficial, morpheaform, infundibulocystic, fibroepithelioma of Pinkus. The most common type is the nodular; it can be easily diagnosed using a handheld RCM device when detection of a single tumor island can be sufficient for a conclusive diagnosis. BCC, when pigmented, shows clearly detectable, bright tumor islands. Superficial BCC is characterized mainly by “streaming” of keratinocytes: ie, the orientation of keratinocytes in the same direction. Dark silhouettes are found mainly in infiltrating BCC.

Superficial BCC is characterized mainly by “streaming” of keratinocytes: ie, the orientation of keratinocytes in the same direction. Dark silhouettes are found mainly in infiltrating BCC.

**Figure 2:**
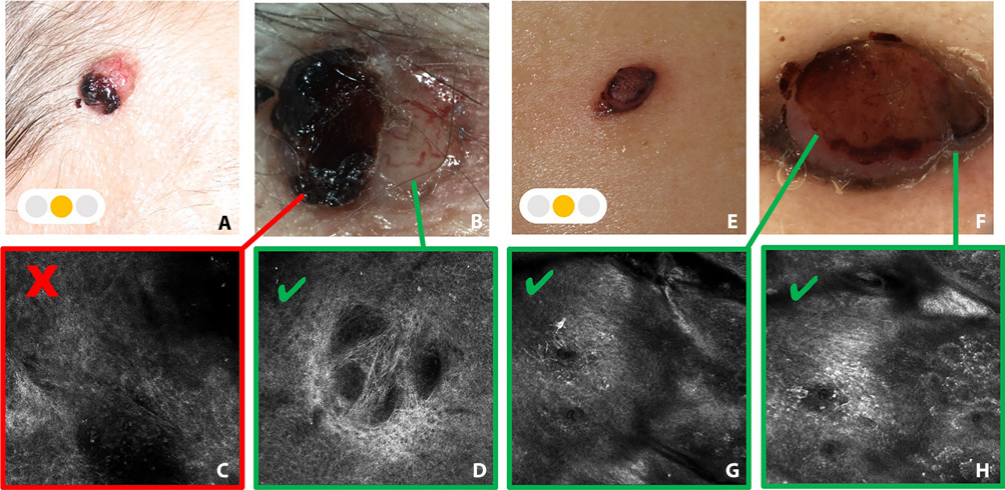
Nodular lesions. (A) Clinical image of a crusted nodular lesion. (B) Dermoscopic image of the crusted basal cell carcinoma (BCC). (C) Reflectance confocal microscopy (RCM) examination does not allow the evaluation of the crusted portion. (D) RCM evaluation of peri-crusted tissue allows the identification of basaloid nest of nodular BCC. (E) Clinical image of the nodular pigmented lesion. (F) Dermoscopic image of the nodular pigmented lesion. (G) RCM examination shows roundish pagetoid cells up-located into the epidermal layer. (H) At the RCM examination in the peri-nodular portion of the lesion, we observe the rings and atypical cells to identify the atypical nodular melanocytic lesion.

A major limit of RCM examination of BCC is represented by the ulceration, bleeding, and crusts, which when present obscure the laser penetration into the tumoral lesion (Figure 2).

Detection of atypical keratinocytes in the context of erythematous-scaling lesions on sun-exposed areas is characteristic of squamous cell proliferations. The histological spectrum from actinic keratosis to squamous cell carcinoma can be studied using RCM with the limit related to the evaluation of the tumoral infiltration into the dermis due to the virtual horizontal sectioning of the tissue and an efficient determination of the grading of atypia of the keratinocytes [23].

### Anatomical Sites

#### Face

The most effective application of the RCM handheld version is in the evaluation of pigmented macules and patches located on the sun-damaged skin of the face [23–25]. The handheld version allows one to reach difficult anatomical sites such as the periocular area, ears, and nose. Moreover, the handheld RCM permits the evaluation of lesions larger than 8 mm in diameter, representing the limit of mosaicking of the static probe. Finally, the handheld device allows, in real time, collection of microscopic data useful for dermoscopy correlation for a quick diagnosis.

Differential diagnosis of pigmented flat lesions of exposed areas comprehends melanocytic skin neoplasm, lentigo maligna (LM), and lentigo maligna melanoma vs nonmelanocytic neoplasms as pigmented actinic keratosis, solar lentigo, and lichen planus-like keratosis [24,26,27]. Dermoscopy diagnosis of pigmented macules of the face remains challenging in some cases when lesions share several dermoscopic features, such as brown pseudonetwork and annular granular structures. In the case of spreading LM cases, handheld RCM supports both diagnoses as well as permits the mapping of the extension of the lesion identifying the margins [28,29]. Several specific features characterizing LM have been reported in the literature (ie, medusa head-like structures, folliculotropism of atypical cells, dendrites in the epidermis, etc) making LM one of the most clear diagnoses on RCM. Differential diagnosis between LM and pigmented actinic keratosis is supported on RCM by distribution around adnexal structures of dendritic, atypical melanocytes [22] (Figure 5).

Superficial and multifocal BCC tumor islands can be difficult to detect on the face because they can be misinterpreted as adnexal structures. In the case of squamous proliferations, especially in sun-damaged skin, atypical keratinocytes are the key for a correct diagnosis. Hair follicles can be differentiated from tumor islands because they are also visible at the skin surface (as hair follicle openings [20,23]).

#### Acral Skin

**Figure 4:**
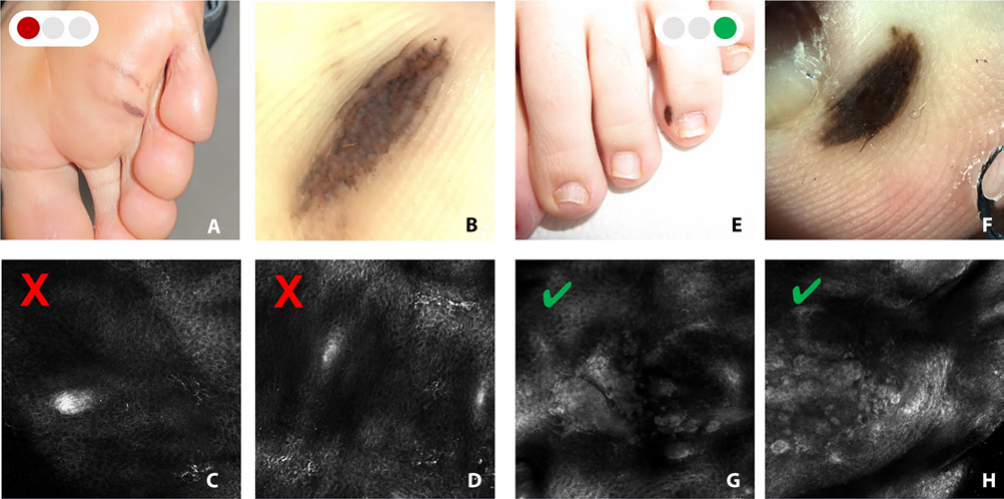
Acral pigmented lesions. (A) Clinical examination of pigmented melanocytic lesion on the sole. (B) Dermoscopic image of an atypical melanocytic lesion. (C,D) Reflectance confocal microscopy (RCM) examination does not allow the evaluation of the melanocytic proliferation due to the hyperkeratosis and acanthosis of the epidermis characterizing the acral skin. (E) Clinical examination of pigmented lesion located at the interdigital space. (F) Dermoscopic image of an atypical melanocytic lesion. (G,H) RCM allows the detection of atypical nests.

**Figure 5:**
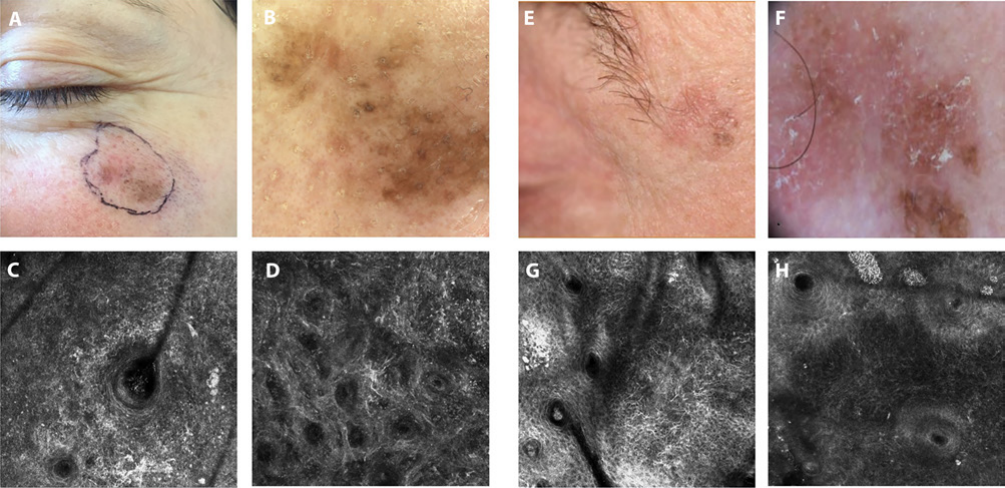
Pigmented macules of the face. (A) Clinical image of a large lentigo maligna (LM). (B) Dermoscopic image of LM showing black rhomboidal structure and obliteration of the hair follicles. (C,D) Reflectance confocal microscopy (RCM) image at the level of the epidermis shows irregular honeycombed pattern and abundant hyperreflective dendritic cells in relation to an ill-defined hair follicle. (E) Clinical image of pigmented actinic keratosis. (F) Dermoscopic image of pigmented actinic keratosis: light brown pseudonetwork and irregular follicular pigmentation. (G,H) RCM image in the epidermal layer shows irregular honeycombed pattern and few fine tangled lines displayed around a well-defined hair follicle without the invasion of the hair follicle.

Normal acral skin is characterized by the presence of ridges and furrows, with the openings of sweat ducts in the center of the ridges, and lack of hair follicles. Epidermal ridges appear as broad parallel bands with a regular honeycomb pattern; epidermal furrows are visible as parallel dark areas disrupting the honeycomb pattern. The openings of sweat ducts appear as bright roundish circles plugged in lines inside the honeycomb pattern. RCM shows Meissner corpuscles as medium reflective ovoid structures in the upper part of dermal papillae, the main touch-pressure sensation receptors in skin without hairs. The dermoepidermal junction (DEJ) and the dermis are not always visible in the acral region, depending on the thickness of the epidermis. In the area around the proximal nail fold near the cuticle and in the fingertips, stellate bright bodies are often visible, corresponding to keratinocyte membranes in a plane not parallel to the microscope tip (Figure 4) [15,30].

#### Oral and Genital Mucosa

Oral cavity and genitalia can be analyzed by RCM to evaluate benign, malignant, and precancerous lesions, identifying specific descriptors for inflammatory or neoplastic diseases [31,32]. Mucosa is particularly suitable for RCM: the absence of cornified superficial layer and the thickness of this epithelium allows for higher definition and deeper penetration of the laser, with no consequent light backscattering and the possibility of a better-detailed visualization of cellular morphology and dermal structures in comparison with the skin tissue. The normal mucosa differs from normal skin in the absence of the stratum corneum and the pigmentation of the DEJ and the difference in keratinocytes details identification: much more detailed in the mucosa with nuclei easily visible in many areas of the upper layers as bright, large, round structures in the center of the cells. In normal conditions, in both oral and genital mucosa epithelial cells are regular in shape and size, differently from malignant tumors, in which the normal architecture is not preserved and cells are not homogeneous [33,34].

The examination of mucosa is limited by the design of the RCM microscopes available in the market, with only possible evaluation of the tissue using the handheld RCM; this device is directly applied through a smaller tip (1.5 cm in diameter) on the genital mucosa or on the dorsal and lateral surface of the tongue, on the inner part of the lips, and in some parts of gingiva. However, for the examination of oral mucosa, characterized by presence of concavity and convexity and mucosal sites not supported by bones and rigid structures, a dedicated handheld device is needed. To reach deeper oral sites with limited accessibility as the floor of the mouth, ventral tongue, gingiva, and inner part of the cheeks, the handheld RCM has been redesigned with a longer optic (approximately 10 cm long and 1 cm in diameter) (Figure 8A).

### Inflammatory Diseases

RCM in the evaluation of inflammatory diseases has been described in the literature to support the clinical-microscopic correlation for diagnosis confirmation and therapeutic management. In detail, a study from the International Confocal Working group demonstrated the role of RCM for the identification of the major groups of superficial inflammatory skin diseases: psoriasiform, spongiotic, and interface dermatitis [5,35,36].

Deep inflammatory processes cannot be examined by RCM because of limits of penetration of laser into the skin.

#### Psoriasiform Dermatitis

##### Prototypic diseases

###### Plaque psoriasis and seborrheic dermatitis

On RCM, psoriasiform dermatitis is characterized by the presence of thickened epidermis (>60–80 μm) and hyperkeratosis (20–40 μm), often in association on RCM with the presence of high refractive round to polygonal structures inside the stratum corneum, corresponding to parakeratosis.

**Figure 6:**
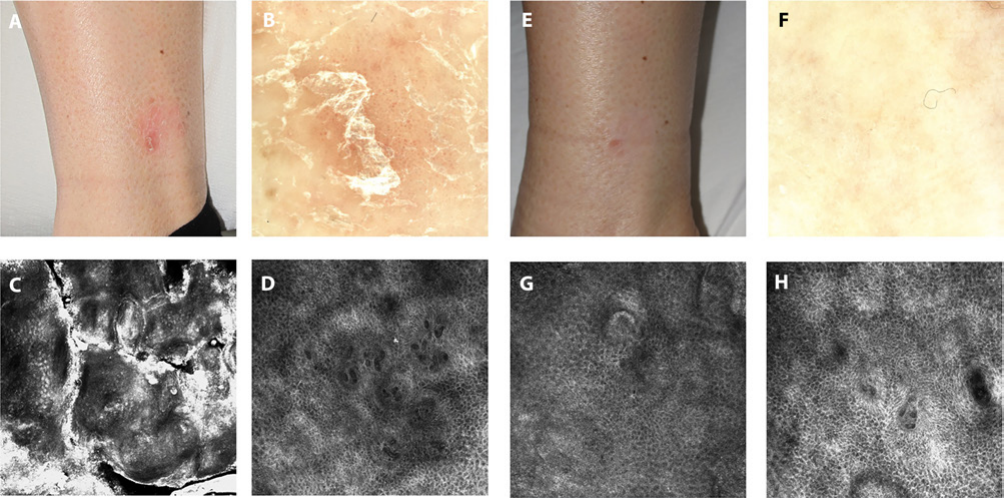
Psoriasiform dermatitis. (A) Clinical image of erythematosus plaque on the leg. (B) Dermoscopic image shows scale and dotted vessel. (C) Reflectance confocal microscopy (RCM) reveals hyperkeratosis and high refractive round to polygonal structures inside the stratum corneum, corresponding to parakeratosis. (D) RCM examination shows enlarged dermal papillae with thin interpapillary epidermal spaces, associated with dilated vessels in a vertical orientation. (E) Clinical image after treatment with local steroid for 3 months. (F) On dermoscopy disappearance of the scale. (G) RCM reveals regular honeycombed of the epidermal layer. (H) RCM shows a decrease of papillomatosis.

On RCM, papillomatosis is seen as “up-located,” enlarged dermal papillae with thin interpapillary epidermal spaces in plaque psoriasis. Prominent dark canalicular structures filling the dermal papillae are detectable, representing dilated vessels in a vertical orientation. The progressive normalization of the aspects can be used for the therapeutic response assessment [37] (Figure 6). Differently in seborrheic dermatitis, dilated vessels are detectable as horizontally oriented and located mainly around adnexal structures; *Demodex folliculorum* located in the ostium of the sebaceous glands represents a specific aspect of seborrheic dermatitis [38].

#### Spongiotic Dermatitis

##### Prototypic diseases

###### Irritant contact dermatitis and allergic contact dermatitis

**Figure 7:**
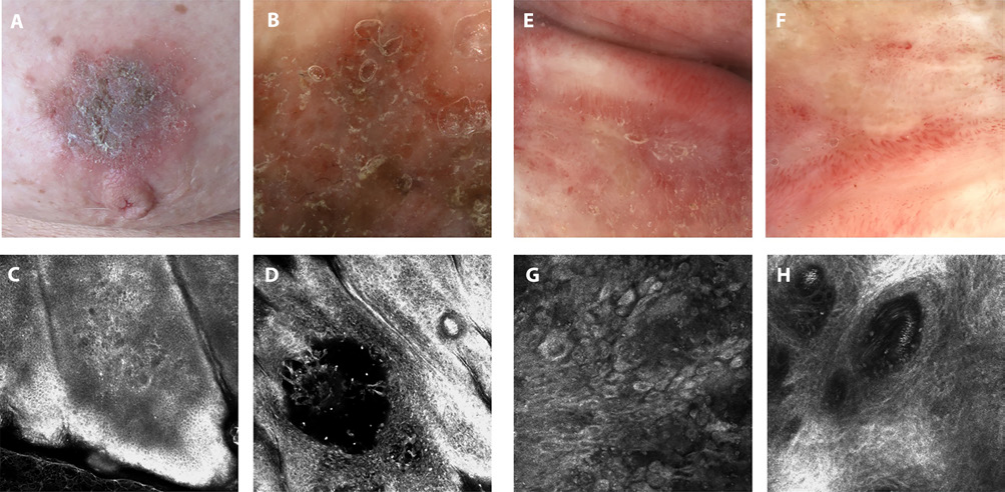
Spongiotic dermatitis and herpes simplex. (A) Clinical image of eczema of the nipple. (B) Dermoscopic examination shows scale and erythema. (C) Reflectance confocal microscopy (RCM) shows intercellular edema and inflammatory cells at the level of epidermis. (D) Vesicles are detected as dark areas at the level of the epidermis, associated with inflammatory cells. (E,F) Dermoscopic evaluation or erythematous tissue of the lip. (G,H) RCM shows atypical keratinocytes with viral cytopathy and intraepidermal vesicles.

Spongiotic dermatitis is characterized by dark areas at the level of the epidermis with broadband intercellular spaces and round to oval bright cellular structures between keratinocyte spaces corresponding to intraepidermal spongiosis associated with the inflammatory cells. In acute phases, intraepidermal vesicles are detected as well-demarcated dark spaces between keratinocytes, filled by “floating” inflammatory cells. The absence of papillomatosis or lichenoid infiltrate, typically observed in psoriasiform dermatitis and interface dermatitis, helps to confirm the clinical suspect of spongiotic dermatitis [35] (Figure 7).

#### Interface Dermatitis

##### Prototypic diseases

###### Lichen planus and discoid lupus erythematosus (DLE)

Interface dermatitis is characterized by the inflammatory process involving the DEJ.

On RCM, the papillary rims are obscured by the presence of the inflammatory cells with an obliteration of the ring-like structures around dermal papillae that are not detectable and not surrounded by the bright rims usually seen in normal skin. Inflammatory cells can be also detected at the level of the epidermis as well as in the upper dermis around vessels and especially around adnexal structures in DLE [38,39].

The handheld version can be moved freely on the skin and allows analysis of large surfaces and of several lesions at different body sites; for these reasons, it is more useful than the static version for the examination of inflammatory diseases.

#### Scalp Diseases

##### Prototypic diseases

###### Scarring alopecia (SA) and nonscarring alopecia (NSA)

Clinical management of alopecia represents one of the major issues in dermatology. Scalp biopsies are not easily accepted, because of the high bleeding and sensitive anatomical area, and are not repeatable during therapy. However, a prompt diagnosis is highly recommended for an early onset of adequate treatment to avoid irreversible hair loss.

In many cases, RCM offers to clinicians the possibility of a real-time, noninvasive distinction between SA and NSA [35,40]. NSA, in particular androgenic alopecia, is characterized by the presence of miniaturization of hair shaft and follicular keratosis. Conversely, the absence of these 2 RCM criteria and the presence of inflammatory cells in the epidermis or around adnexal structures are suggestive of SA (lichen planopilaris, DLE, other). SA is classified as interface dermatitis, characterized by involvement of the DEJ, with consequent obscuration and disappearing of the dermal papillae and absence of the normal rimming at the RCM examination. Infiltration of adnexal structures represents the main expression of the interface change, and in lichen planopilaris it can be the prevalent location of the inflammatory cells’ infiltration [41]. With RCM, dermatologists involved in the management of scalp disease can assess the amount of inflammatory cells and evaluate the effective status of the disease’s activity.

### Infectious Diseases

Several descriptions of RCM features of cutaneous parasites, such as *Demodex folliculorum* and *Sarcoptes scabiei*, and infectious skin diseases have been reported in the literature, and there have been sporadic reports of detection of dermatophytes using RCM [7]. However, probably the most effective application of RCM in infectious diseases is in viral diseases. Even if RCM cannot directly observe virus, cytopathic effect associated with herpes viruses such as varicella zoster and herpes simplex has been described, underlining the possibility of a prevesicular stage detection [42]. Compared to blood tests and cytological direct tests, RCM has the advantages of being noninvasive, quick, and able to image the entire affected cutaneous surface.

### Therapeutic Follow-up and Biopsy Site Selection

**Figure 8:**
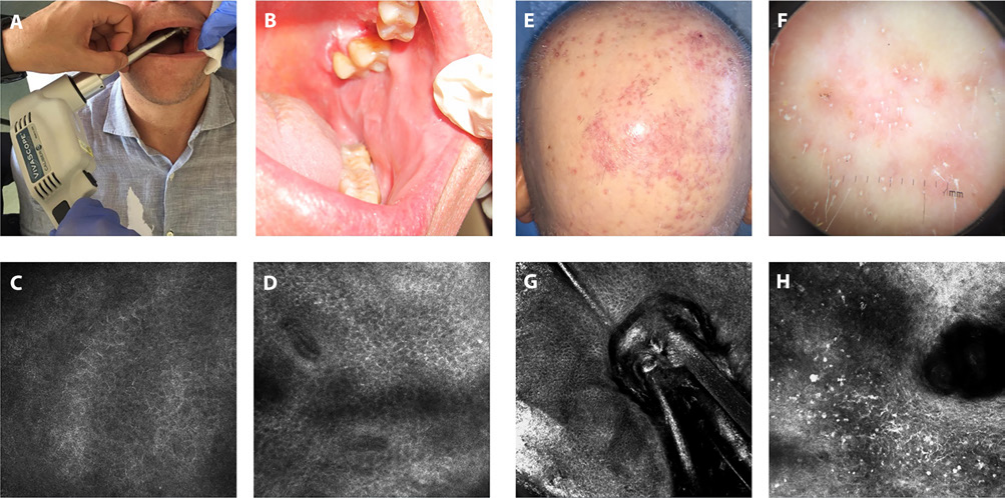
Interface dermatitis. (A) Clinical image of the use of the new probe, specific for the oral cavity. (B) Clinical examination of lichen in the inner part of the cheek. (C,D) Reflectance confocal microscopy (RCM) examination. In oral lichen, inflammatory cells in the epidermal layer are detectable. (E) Clinical examination of scarring alopecia (lichen planopilaris). (F) Dermoscopic image shows white areas and follicular hyperkeratosis. (G,H) RCM shows the absence of miniaturization of hair shaft and follicular keratosis and the presence of inflammatory cells in the epidermis or around adnexal structures and the consequent disappearing of the normal rimming.

Once RCM descriptive parameters of a specific neoplastic or inflammatory entity are defined, the normalization of the features during medical treatments can be used for the therapeutic response assessment (Figure 6). Therapeutic follow-up has been reported in the literature for psoriasis, cutaneous lymphomas, noninvasive therapies for nonmelanoma skin cancer, and scalp diseases [43–46] (Figure 8).

Moreover, in specific diseases (mycosis fungoides or scalp alopecias), or in patients with multiple lesions (lichen planus, lupus), RCM imaging can be used as a real-time guide for optimal biopsy site selection to reduce the number of unsuccessful histopathological examinations or to avoid late-stage lesion sampling [35,40].

## Conclusions

RCM is a high-resolution, noninvasive imaging technique used in the diagnosis of both melanoma and nonmelanoma skin tumors and in the interpretation and management of inflammatory skin diseases.

Two different devices with different designs for specific indications are available in the market: a static and a handheld probe. The typical limits are represented by hyperkeratotic and ulcerated lesions. In acral sites, RCM can be used only in a limited number of cases. We describe the indications for the use of RCM for a schematic approach for practical purposes.

## Figures and Tables

**Figure 1:**
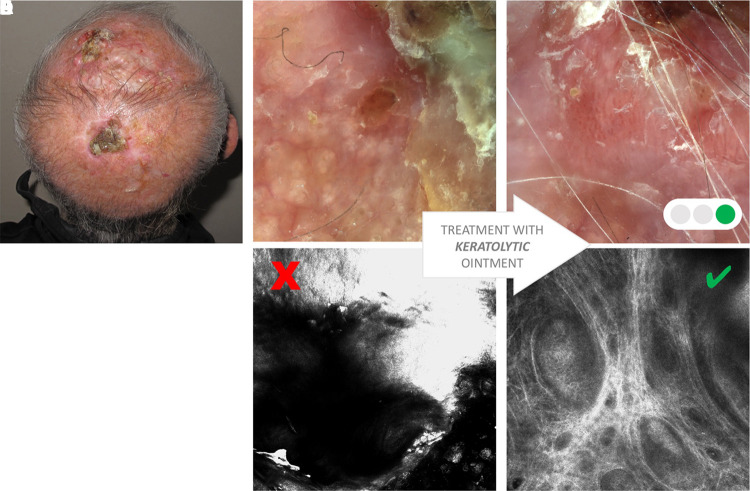
Hyperkeratotic lesions. (A) Clinical presentation of hyperkeratotic lesion on the scalp. (B) Dermoscopic image of the hyperkeratotic portion. (C) Reflectance confocal microscopy (RCM) examination does not allow the evaluation of the hyperkeratotic portion because of high backscattering of the light due to keratin. (D) Dermoscopic image of the same area after 2 weeks of keratolytic ointment application. (E) RCM examination allows identification of basaloid nests of basal cell carcinoma.

**Figure 3:**
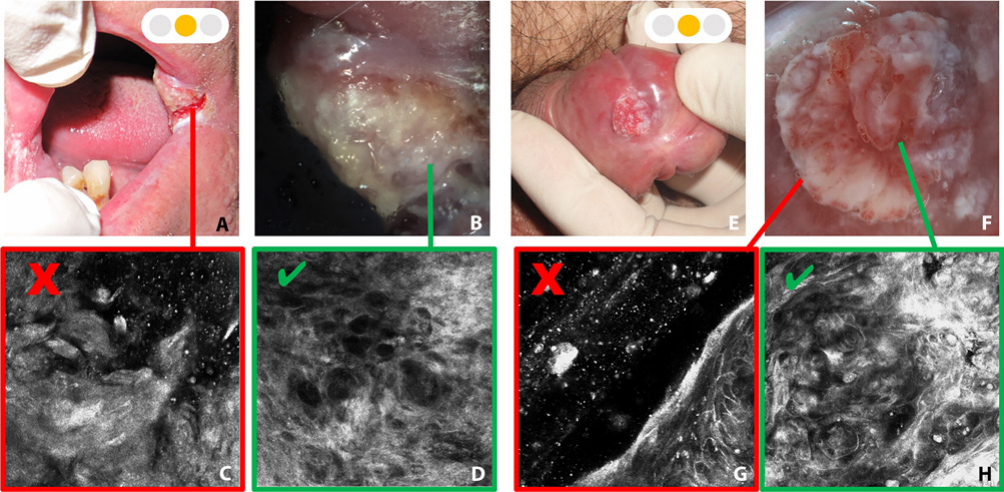
Ulcerated lesions. (A) Clinical presentation of oral squamous cell carcinoma (SCC). (B) High magnification image of the SCC located on the labial commissure. (C) Reflectance confocal microscopy (RCM) examination does not allow the evaluation of the ulcerated portion. (D) RCM shows severe cellular atypia of the epithelium in the peri-ulcerated portion. (E) Clinical examination of vegetative ulcerated lesion of the glans. (F) Dermoscopic image shows white concentric structures as SCC. (G) RCM examination does not allow the evaluation of the peripheral portion. (H) RCM of the central portion is characterized by architectural disarray and cellular pleomorphism at spinous and granular layers as sign of the cellular severe dysplasia of SCC.

**Table 1:**
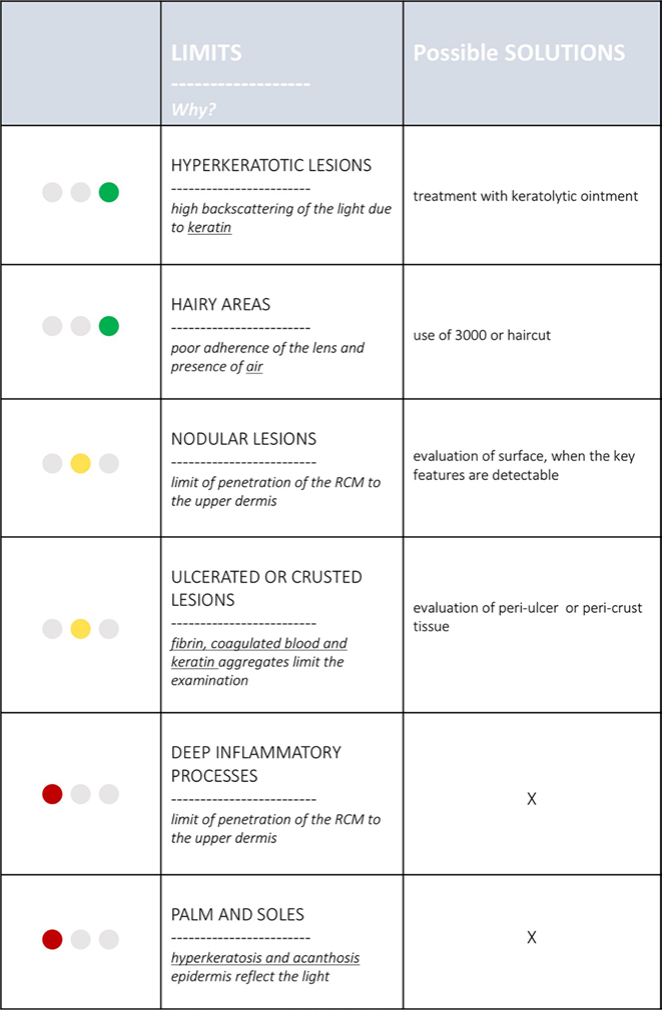
Limitations of Reflectance Confocal Microscopy (RCM) and Their Possible Solutions

Green = possible to overcome; yellow = not always easy to overcome; red = never overcome (Figures 1–4).
